# Qualitative evaluation of a social media campaign to improve healthy food habits among urban adolescent females in Indonesia

**DOI:** 10.1017/S1368980020002992

**Published:** 2021-06

**Authors:** Pande Putu Januraga, Doddy Izwardi, Yessi Crosita, Putu Ayu Indrayathi, Eny Kurniasari, Aang Sutrisna, Alison Tumilowicz

**Affiliations:** 1 Faculty of Medicine, Center for Public Health Innovation, Udayana University, Bali, Indonesia; 2 Kementerian Kesehatan Republik Indonesia, Jakarta, Indonesia; 3 Global Alliance for Improved Nutrition (GAIN), Jakarta 12950, Indonesia; 4 Global Alliance for Improved Nutrition (GAIN), Geneva, Switzerland

**Keywords:** Foods, Social media, Nutrition campaign, Healthy eating, Adolescent girls, Indonesia

## Abstract

**Objective:**

The current study focuses on how adolescent girls in urban Indonesia accept technology in a social media (SM) campaign to promote healthy eating habits.

**Design:**

The study was a qualitative evaluation of the online campaign. In-depth interviews using semi-structured interview guidelines and focus group discussions were used to collect data. Data were analysed using a general inductive approach to provide simple and straightforward answers to our study questions.

**Settings:**

The study was conducted in two urban areas in Indonesia: Jakarta and Jogjakarta.

**Participants:**

Adolescent girls aged 16–19 years.

**Results:**

The SM campaign was perceived as beneficial for increasing participants’ knowledge. The campaign helped increase participants’ awareness of healthy diets and the health risks of unhealthy diets as well as increase their motivation to change their behaviour and avoid foods containing salt, sugar and excess fat. The participants perceived information from the online campaign as complete and trustworthy. Instagram was cited as the easiest platform to use, while the website was cited as having the most complete information. YouTube provided the best viewing experience but was considered a data-heavy platform. The barriers to change were perceptions of taste, limited choices for healthy but affordable ingredients and family-related factors.

**Conclusions:**

The online nutrition campaign was well accepted by Indonesian urban adolescent females and motivated them to act to protect their health. Future nutrition-related SM campaigns aimed at this demographic should focus on platforms with the greatest benefit and ease of use.

Adolescents are vulnerable to malnutrition, which leads to health problems such as obesity and anaemia^(1)^. Moreover, the nutritional and health status of adolescents, especially adolescent girls, has an intergenerational impact. The health and nutrition of adolescent girls are important determinants of health both for childbearing females and for their infants^(2,3)^. Indonesia, similar to many other developing countries, is facing a double burden of malnutrition – a combination of under- and overnutrition – coexisting in the same population, including in adolescent girls. The Indonesian Basic Health Survey estimated that nearly half of all non-pregnant girls aged 15–19 years were suffering from chronic energy deficiencies and that approximately a quarter of 15- to 24-year-old women were suffering from anaemia, while a third of women over 18 years were overweight or obese^(4)^.

A high intake of fast foods, soft drinks and processed foods as well as a lack of physical activity has contributed to female adolescent obesity in Indonesia, especially in urban areas and among young women with a higher socio-economic status^(5,6)^. On the other hand, low consumption of animal-sourced foods, a low energetic intake and poor snacking habits have led to anaemia among a sizeable number of Indonesian adolescent females^(7)^.

In the last decade, adolescents in many countries have been living in a media-saturated environment, and Indonesian youth are no exception. A survey carried out by the Association of Internet Service Providers in Indonesia in November 2016 showed that Internet penetration in Indonesia has now reached 51·8 % of the population, constituting 132·7 million Internet users in Indonesia. The survey also found that 75·5 % (24·4 million) of Indonesian youth aged 10–24 years were Internet users, with the most common device used by all users to access the Internet being the mobile phone (63·1 million users)^(8)^. The number of current, active Instagram users in Indonesia has more than doubled since 2016, and compared with Instagram users from other countries, Indonesian users are among the most prolific story content producers worldwide^(9)^. In Indonesia, Facebook remains the most popular social media (SM) platform, with 87·75 million users^(10)^, and its popularity is higher among older users (aged 30–35 years), while Instagram is more popular among users aged 16–25 years^(11)^.

The changing Internet ecosystem has enabled active participation by users, allowing them to connect, interact and exchange information on topics of interest, such as healthy eating habits, and to build supportive communities^(12)^. In addition, systematic reviews have shown positive results concerning the use of SM in health promotion campaigns in the field of nutrition^(13)^.

Against the backdrop of the wide popularity of SM use among urban adolescent girls in Indonesia^(14)^ and positive prior results for using SM for health and nutrition education, the Global Alliance for Improved Nutrition Indonesia and its partners developed an online and offline campaign to promote nutritious food choices among urban, unmarried adolescent girls aged 16–19 years. The SM component of the campaign, named *Pretty and Picky*, used the hashtag ‘**#**ThePrettyandPicky’.

Nutrition-related online campaign strategies are relatively new to the Indonesian audience, particularly among adolescent girls. Thus, an evaluation of this strategy is needed to better understand the acceptance of the campaign by adolescents. A better understanding of users’ experience and acceptance is expected to inform future strategies in behavioural change models as the piloted campaign is scaled up. The technology acceptance model (TAM) is one of the most prominent behaviour models to explain why users accept or reject health information technology^(15,16)^. By exploring participants’ perceptions and experiences in accessing and utilising online campaign platforms, the current paper aimed to apply TAM frameworks to understand participants’ acceptance of the new online campaign strategy. In the current paper, we report the results of the qualitative evaluation of the *Pretty and Picky* online campaign.

## Methods

### *The Pretty and Picky* campaign components

The campaign was tailored to young, unmarried Indonesian girls’ personal characteristics and preferences. It was designed based on the notion that the daily practice of choosing healthier food is reinforced by increased knowledge of nutrition and enhanced awareness of the importance of healthy eating, as well as by motivation, social supports and the provision of practical tools. The fundamental thesis of the multiplatform SM campaign implemented in *The Pretty and Picky* campaign was that to influence social norms, key messages needed to be widely conveyed, discussed and shared in the adolescent girls’ network in order to shape socially acceptable behaviour^(17,18)^.

The key messages for the SM-based campaign were grouped into several specific themes: (i) increasing fruit, vegetable and water intake, (ii) reducing sugar, salt and fat intake and (iii) assessing food composition and labels. The campaign involved two core strategies: the first was the online, SM programme as the heart of the campaign, and the second was an offline programme to support the online component. The following SM hashtags were used: #JanganAsalPilih (choose wisely), #JanganAsalGalak (reduce sugar and salt), #JanganAsalComot (do not eat carelessly) and #KepoinKomposisi (pay attention to food composition). The campaign encouraged conversations about healthy eating choices, promoted the sharing of healthy food with peers and educated audiences using fun and engaging SM activities such as video creation and contest participation. On all the campaign’s SM platforms, there was a link to the campaign’s webpage.

*The Pretty and Picky* website provided information for teenagers on recent articles about healthy eating, Instagram content and a BMI calculator. The website was also linked to content promoting healthy food on Facebook, Instagram, YouTube and LINE and vice versa. In general, *The Pretty and Picky* campaign provided teenagers with activities, articles, recipes and photos related to healthy food choices. The article section provided adolescents with helpful tips and information about the teenage years, lifestyle, food, beauty and health, while the recipe section gave visitors access to simple and healthy recipes in video and photo formats. Offline programmes educating adolescents about healthy food choices were also conducted to promote the online campaign.

### Theoretical framework of the qualitative evaluation

The campaign objective was to promote healthier food choices among adolescent girls in Indonesia, using SM as a novel approach. Our study question was, ‘How does our target audience accept the campaign?’. This question was considered too broad for quantitative research; thus, a qualitative study using a general inductive model was chosen to provide simple and straightforward answers to the question. Furthermore, the TAM was chosen to help guide the evaluation, since this model is commonly used to evaluate consumers’ acceptance of information technology, particularly computers^(16)^. The TAM was first published as a PhD thesis in 1985^(19)^. The theory assumes that the intention to use and the actual use (acceptance) of a new information system/technology depend on two major factors, which are its perceived usefulness and its perceived ease of use. Since its development, the TAM has been the most widely applied theoretical model in the field of information systems^(15)^. The use of TAM as a theoretical framework has expanded to different technologies (including health information technology), situations and settings, with different controlled factors, such as gender, age, education and organisation^(15)^.

In the field of health promotion, the TAM has mostly been used to evaluate consumers’ behavioural intentions to use health information technology^(20–23)^. Some studies have integrated the TAM with other popular theoretical frameworks in the health promotion field, such as the health belief model^(20,23)^, and most studies have emphasised quantitative methods, as introduced by the original TAM study. In general, quantitative studies have aimed to test associations between constructs and variables, allowing researchers to investigate a known relationship in greater detail^(15,24)^. On the other side, there has been a lack of qualitative approaches to explore how settings and backgrounds influence complex interactions between humans and technology^(24)^. This has been the main criticism of the TAM, which heavily focuses on quantitative research methods.

As a result of this gap, more studies have applied qualitative methods to study technology acceptance^(24,25)^. Some examples of the use of TAM in qualitative approaches are a case study of the public acceptance of renewable energy^(26)^ and another case study that explored perceptions of home telemonitoring use in patients with long-term conditions^(27)^. Both studies explored perceived usefulness and ease of use as mediating factors of technology acceptance.

As our SM-based campaign provided a wide range of information, it was important to explore aspects of the perceived usefulness of the intervention, such as what content was considered useful and why and how this content matched the needs of our target audience. In addition, our intervention was delivered through multiple SM platforms. Thus, exploring the perceived ease of use of each platform and the reasons for any identified user preferences are also crucial for designing future interventions aimed at this demographic. However, since our respondents were already users, instead of exploring the intention to use as indicated in the initial TAM, we explored usage behaviour. Furthermore, we explored the link between the exposure to our campaign and the intended behaviour change promoted by our intervention. Hence, a qualitative study employing a general inductive approach with a modified TAM as a guideline was more appropriate to answer the study question.

### Study design, participants and data collection

After the online campaign was implemented in May 2017, a qualitative assessment was conducted to document and analyse programme participants’ acceptance of the SM campaign. Compared with other approaches, a descriptive and observational research design using a qualitative method can yield richer information, allowing for the evaluation of the programme from the participants’ point of view^(28)^.

We conducted semi-structured, in-depth interviews with thirty-seven 16- to 19-year-old girls from two major cities in Indonesia (Jakarta and Yogyakarta) who were online campaign participants. Respondents of the in-depth interviews were identified through their engagement with our SM campaign and were recruited via open, online recruitment through the SM platform.

During the 2 months of data collection, twenty-five respondents in Jakarta and twelve respondents in Yogyakarta agreed to participate. The research team then set the time and place for the interviews. The number of subjects in Yogyakarta was less than the proposed number due to recruitment difficulties, as an insufficient number of girls responded to the interview invitation that was shared via the campaign. Initially, it was decided that informants should be selected purposively based on the SM platforms they accessed. However, due to the limited number of respondents who confirmed their participation in interviews, all candidates were recruited as research informants. From the list of informants willing to be interviewed, most had accessed multiple SM platforms, with Instagram being the most frequently accessed platform. To have saturated data on the acceptance of the online campaign, we also conducted three focus group discussions (FGD) with students (ten students per FGD) who had participated in roadshows and offline campaigns at two schools in Jakarta: senior high schools (Sekolah Menengah Umum (SMU)) thirty-six and seventy-eight. Since the focus of the evaluation was the online campaign, participation in the online campaign was the main criteria for the FGD participants’ recruitment. The other criteria were the same as those for the in-depth interview recruitment.

The in-depth interviews were conducted by trained enumerators in mutually agreed-upon locations, with each interview lasting 40–90 min. The FGD were conducted at each school. All interviews were conducted in Bahasa Indonesia, and the audio was recorded. The questions for the in-depth interviews and FGD were guided by the TAM domains of perceived usefulness and perceived ease of use of the online campaign platform in relation to content and usage behaviour.

### Data analyses

Data were analysed using the general inductive approach for qualitative data. This method is beneficial as it is a simple, straightforward approach for deriving tangible findings from the data gathered based on evaluation questions. The evaluation objectives provide a focus (or domain of relevance) for conducting the analysis rather than a set of expectations about specific findings^(29)^.

The qualitative data analysis software NVivo version 11 (QSR International) was used to manage the transcripts and process the analysis. First, the analysis procedure began with reading the interview transcript carefully to gain an overall impression of the participant’s perspective of the campaign. Next, we conducted open coding to summarise the text or a part of a sentence(s) from the transcript. This produced a large number of codes (or nodes) for specific themes relevant to the acceptance of the SM campaign; for example, nodes were produced for ‘cross platform’, ‘easy to navigate’, ‘memorability’, ‘trustworthiness’, ‘healthy food is not delicious’, etc. Next, using NVivo and axial coding^(30)^, the nodes were grouped into categories as follows: health information, environmental factors, technology and behaviour. With the current paper’s evaluation objectives in mind, the nodes within each group were then selectively assigned to the major themes based on the adapted TAM: perceived usefulness, perceived ease of use and usage behaviour as well as new themes that were grounded in the data transcripts. All analyses were performed in Bahasa Indonesia, and the relevant quotes were then translated into English.

## Results

This section describes the five major themes or categories identified from the data analysis, which were as follows: (i) the perceived usefulness of the SM campaign content, (ii) the perceived ease of use of the SM campaign platform, (iii) usage behaviour in the SM campaign, (iv) intention for behavioural change as a result of exposure to the SM campaign and (v) barriers to behavioural change and suggestions for campaign improvement. The themes generally reflect findings that were observed on multiple occasions or that strongly represent the dimensions of acceptance. We present excerpts from the transcripts to represent the findings.

### Perceived usefulness of the social media campaign content

The perceived usefulness of the SM campaign was largely related to its content in the health information group based on the axial coding, and it was especially related to the quality of the information, such as its trustworthiness and the simplicity of messages, in the original nodes. The qualitative exploration of user engagement indicated that many respondents were engaged by the content offered by the SM campaign and often liked the message and shared it both online with their network and offline with their friends and family. The messages conveyed regarding healthy eating habits were found to be useful, because they often provided new knowledge and practical information for respondents.

I like the content from the nutritionist because they are the expert, and the way they explained it is very clear, like when it is said that we need to reduce sugar and she said it means only one donut a day is allowed, I was like ooo ya now I know. (FGD 1)

Through their engagement in the SM campaign, respondents demonstrated an increased awareness of unhealthy eating habits and of the risks associated with type 2 diabetes, hypertension and cardiovascular disorders.

Don’t just eat snacks on the street, a lot of oil, a lot of salt, iced drinks contain a lot of sugar. Also, the #Jangan AsalGalak post talks about the three elements of sugar, salt, and fat. Well, also we cannot have a lot of salt, sugar, or fat because it causes the diseases that I mentioned before (hypertension, kidney problem, diabetes). Now I am aware of that information. (R6)

Further, regarding the topic of messages in the campaign and the language used to package health content on the site, the participants’ impression was that it was easy to understand, clear, simple and age-appropriate. The terms often used to describe the site’s language clarity were ‘not too high’ or ‘not heavy’. This is a vital finding, as message clarity is important for improving the effectiveness of a health education campaign.

It uses language that is not too high, yes, maybe that is about my standard. We read it and can easily understand it. It is simple. So, whoever reads it can “get it”. (R23)

In addition, respondents also reported gaining a better understanding of the importance of healthy behaviours that are easy to adopt and have a major impact on health but are often forgotten; examples of this include drinking eight glasses of water a day and reducing the consumption of junk food and fried food.

I didn’t realize that we should drink eight glasses [of water] per day before, now I do. (R2)

Other knowledge gained by participants during the campaign was related to BMI. Participants felt that the interactive BMI calculator was an added value to their campaign participation.

### Perceived ease of use of the social media campaign platform and usage behaviour

The perceived ease of use of the SM campaign mainly originated from the technology and behaviour group in the axial coding and was mostly related to having good visual presentation, not being data heavy and having complete information in the initial nodes. The campaign’s information about beauty and health was presented in a unified manner, eliminating the need to search for missing information, which can be difficult and often lead to conflicting results. The complete and specific information that was offered encouraged users to form positive perceptions and to trust the veracity of the information. In summary, a recipient’s belief in the validity of the information provided in an educational programme is a particularly important factor in determining the success of the programme.

For example, on Google, if we search for nutritional info or whatever, we get lots of search results from everywhere and the longer we look, the more confused we get. But with Pretty and Picky, we get one page which focuses on nutrition so whatever we are looking for, everything is right there. (R7)

Regarding the ease of use of each platform, respondents cited that accessing Instagram was easier and that it provided a stronger visual impression of the content, making it more attractive to teenagers. Furthermore, the content on Instagram was concise and easier to understand and remember; thus, it appealed to teenagers. Another feature of Instagram that appeals to its users is its ability not only to display interesting images but also to host short videos and status updates as well as messaging.

Umm, it explains it bit by bit, for example, like on Instagram, it only has photos and only a short but clear explanation, so I understand it easily. (R2)

However, regarding platforms’ completeness, compared with the other platforms, the website was viewed by participants as providing more health tips and access to a larger body of information about healthy food. The website also allowed users to select specific kinds of information or menus. Another appealing feature of the website was the interactive BMI calculator, which enabled users to discover dietary information and activities that matched their own individual needs.

It’s great. When we go onto the website, it asks us what our name is, our age, our weight, our height. Then, it tells us if we are at our ideal weight or not. It also recommends what we should eat for lunch and dinner and what activities we should do. (FGD 2)

However, Facebook was perceived as ‘not for our generation’. The research participants found that Facebook’s popularity had declined, with many claiming it was ‘starting to be abandoned’, ‘rarely used’ or ‘already quiet’. In fact, some participants claimed to access #ThePrettyAndPicky via their mother’s Facebook accounts. The same was true for LINE; many respondents considered this platform not to be popular anymore.

YouTube received a favourable response from the participants. Many claimed that YouTube provided the best video-viewing experience related to preparing healthy food (using healthy recipes). However, YouTube was considered to be a *data-heavy* platform by the study participants, who as teenagers still relied on parental support for Internet access. Thus, some participants expressed reluctance to regularly access the campaign via YouTube, or they chose not to use YouTube at all.

Another aspect of the campaign that positively affected its likability was its cross-platform design. This feature allowed users to switch between SM platforms according to their needs. For example, from the website, visitors who needed more detailed information on a recipe could switch to YouTube. For Instagram users, the link to the website was provided in the bio so that users could access it if they required more detailed information on health tips. A bit.ly link to the specific post in the website was also provided in the caption.

Yes. Because I am so curious about health, so I like to search here and there, so if I open the first one and there is not enough info there, I just open the (The Picky and Pretty) website. (R22)

### Intention for behaviour change as a result of exposure to the social media campaign

According to respondents, the most important improvement in their motivation during the campaign was their intention to initiate behavioural change earlier rather than later. Their change in attitudes towards healthier food selection was closely related to their increased knowledge of the risks from eating unhealthy food. In addition, the knowledge they acquired from the campaign about healthy foods and meals that they could easily access or prepare themselves also motivated them.

Well, that’s what I am, because we are heading towards our 20s so I’m now just more aware of my health, so I’m not waiting until I get old to prevent it [ill health] so I am preventing it now, for example, by having what is good for my skin, my body. (R6)

The foods [presented on the website] are so much better…compared to other food, nicer and healthier than other “healthy food”. (R6)

Another factor closely related to the intention to adopt healthier behaviour was the perception of risks and the potential impact of unhealthy foods on youthful female beauty. For participants, beauty was a powerful motivation for improving their lifestyle choices. The #theprettyandpicky campaign was recognised by many participants as promoting the idea that choosing and eating healthy food would have beauty benefits.

Because it is beauty, for teenagers now, who does not want to be beautiful, right? Who does not want to be healthy? This will definitely make you look beautiful too. For example, if fat causes stretch marks, it is not good, why do we want to have stretch marks? Teenagers have stretch marks. That is not good, right? And health is for our future. I don’t want to be old and sick, so I’ll start making a difference now. (R7)

However, when probed further on the topic of beauty and nutrition, many participants preferred a more positive framing of the message.

It should be about healthy lifestyle, not focusing on the ideal body shape. Girls our age, 17, most of them want to have an ideal body but of course it depends on our eating habit and our lifestyle. So, I think we need to say more about that and not being fat is just a bonus to being healthy. (FGD 1)

Being comfortable with yourself is more important, like when someone is fat but they feel OK it should be OK so the message should be about eating healthy and healthy lifestyle. (FGD 1)

In addition to being motivated by beauty benefits, participants were more motivated because of their new perception that healthy food can also be delicious. The campaign, which included information on foods that were healthy, tasty and easy to prepare, convinced the participants that healthy food is delicious and simple to make, which enhanced their self-efficacy.

In the in-depth interviews, some examples of participants’ actions that represented actual behavioural change were recorded. Specifically, participants cited changes in the way they selected food to avoid excess salt, sugar and fat, to increase fibre intake by eating fruits and vegetables and to drink more water. This change in dietary patterns was linked to an increased understanding of the risks that excess sugar, salt and fat present, as well as the need to drink eight glasses of water per day. This effect can be attributed to the efficacy of the garam, gula, lemak (GGL) (sugar, salt and fat) message.

Before, I ate haphazardly, but after I knew [about healthy eating] I began to choose more carefully. So, when I was eating something healthy, I was like “Oh wow, actually it’s really tasty.” I never imagined that healthy food could be so delicious. (R3)

When I am hanging out with my friends, I tried some food suggested by the website. My friends ordered hot chocolate, chocolate milkshake but I ordered plain tea with no sugar and it was actually OK. The next day I ordered it again and my friends ordered low-sugar drinks too, which was unusual. I want to live a healthy life now by reducing my sugar intake. My friends are also joining in and I don’t think it is that hard. (R6)

In some cases, the behaviour changes involving reducing or avoiding foods containing salt, sugar and excess fat appear to have encouraged peers to do the same. Another positive behaviour of participants after their exposure to the campaign was attempting to influence parents (especially mothers) to cook healthier meals and participants, themselves, trying to prepare healthy meals using recipes from #theprettyandpicky. Some participants reported that as a result of the knowledge gained from the SM campaign, they dared to argue with their mothers, who may not have been attentive to the health impacts of their daily choices about ingredients and food preparation. Other participants reported having had opportunities to experiment with recipes from #theprettyandpicky campaign. Although not for most participants, this was nevertheless a positive outcome for some that could provide lessons for future improvements of the campaign – for example, in the selection of recipes for campaign materials.

### Barriers to behaviour change and suggestions for campaign improvement

As noted by the study participants, the most prominent individual-level barrier to participants making healthy food themselves was the persistently strong perception that healthy food is not as tasty as foods containing high levels of salt, sugar and fat. Although some participants began to realise that healthy foods can be interesting and flavourful, several participants continued to believe that unhealthy foods were ‘tastier’ than healthy ones.

I can’t resist the temptation of MSG and sugar, it’s like my taste buds are already used to it. (R1)

In addition, the target audience’s demanding academic schedules and their lack of cooking skills meant that they may not have had the time to experiment with the recipes suggested by the campaign. Thus, the perception that healthy food is not particularly tasty persisted.

Regardless of the campaign’s strategy to accommodate these factors by presenting various fast and easy-to-make recipes, some participants still encountered structural obstacles, such as living conditions with limited cooking facilities.

Yeah, I only just found out the information, but to put it into practice, I just don’t have time for that just yet. (R6)

Hehe, I’m like a boarding-school kid, I only brought a rice cooker. Maybe I can cook them [the recipes] with a rice cooker? (R30)

Gathering feedback on recipes that can be adapted for simple, widely available cooking tools such as those in boarding houses and similar living environments is important if the campaign wishes to encourage a wider adoption of healthy eating habits. In addition, in terms of the difficulties of following the recipes presented by #theprettyandpicky, participants pointed out the importance of providing widely available and inexpensive alternative ingredients. Many young women involved in the study were not accustomed to shopping in grocery stores and traditional markets, as the price of the ingredients in these supermarkets was considered more expensive. The high price of ingredients on top of the time and effort needed to cook the healthy meals was discouraging to some participants.

I like the recipe, it’s easy to make but I need the information on different ingredients that are cheaper and easy to find but of course healthy. It is also important to introduce alternatives for salt and sugar; we don’t like to eat bland food, right? (R29)

Personal barriers, such as a lack of perceived self-efficacy, and interpersonal barriers, such as peer pressure and the lack of parental support for healthy eating habits, were identified. The identified environmental barriers to change were related to the availability of healthy food at home and in the school canteen as well as to a lack of control over food at home or when going out with friends.

Participants reported difficulty behaving consistently choosing healthier foods or beverages in group situations because there is a tendency to choose based on group preferences and to support the atmosphere of *having fun*.

For example, like hang-out food, it is more like junk food, isn’t it? It’s so difficult to say no. (R17)

Participants also cited their dependence on dishes prepared by parents together with the Eastern value of appreciating home-cooked food, which convinced them they had no other choice and could not argue with parents about healthy food choices.

My packed lunch consists of mainly fried food. Nuggets that are easy to fry really. Because it is hard to wrap up vegetables, so they do not spill on the way to school. So usually, yeah, nuggets, sausages, potato, stuff like that and I must eat it anyway. (R14)

## Discussion

In the current study, we found indications that the campaign was well accepted by Indonesian urban adolescent females and that it motivated them to act to protect their health. The study also documented several lessons learned, including the need for more focused and frequent messages and to select communication channels emphasising the platform with the greatest ease of use and usefulness to improve the campaign’s reach and level of engagement.

Very few studies have been published that utilised qualitative approaches in evaluating online, health-related campaigns. We believe this is the first study in the Asia Pacific region to document the qualitative evaluation of an online, cross-platform campaign designed to promote healthy eating choices among adolescent girls. Most healthy eating campaigns targeting adolescents have employed quantitative methods, such as a randomised clinical trial testing the impact of a website promoting nutrition and physical activity for adolescents^(31)^. In addition, the Butterfly Girls study in the USA, which promoted a healthy diet and physical activity, was evaluated quantitatively^(32)^.

Another key strength of our study was using the TAM as a theoretical framework to help guide our exploration. In most quantitative studies employing the TAM framework, the intention was to test the theory itself and whether it fits the study constructs or model. Moreover, in a qualitative approach, the theory has also been used to develop new constructs of acceptance^(24)^. One review stated that the TAM is useful but must be integrated with constructs that explain human and social change processes^(33)^. In a study of public acceptance of renewable energies that incorporated the TAM framework and qualitative data collection, the relevance of structural factors such as company commitment, the general public’s participation and location choice was revealed as important to acceptance^(26)^. In the current study, we present not only perceived usefulness and perceived ease of use but also usage behaviour and individual and environmental factors that influence the acceptance of the campaign. These are discussed as follows.

Concerning the choice of campaign channel, based on the reach and engagement data and the qualitative explorations, Instagram occupies a unique position among young, female SM users in Indonesia, allowing access to YouTube and other SM platforms through a link provided in the bio or caption. This finding is in line with the Instagram internal analytic data showing that Indonesia has approximately 42 million active users per month, the largest market in the Asia-Pacific region^(17)^. However, cross-platform interaction in Instagram is not as easy as it is in Facebook. If users want to go to a website, they must scroll back to the bio or copy and paste or type the caption’s link in their browser.

Our findings suggest that adolescent girls enjoyed the cross-platform design of the online campaign, with different apps used to deliver the messages. Cross-platform designs that use multiple SM platforms simultaneously, such as Instagram, YouTube, Website, LINE, Facebook and Blackberry Messenger, do allow greater reach but may be less effective because of dispersion of the audience across different platforms without meaningful engagement^(34)^. An effective healthy eating habit of SM campaign must segment its priority populations, and this involves identifying the target population’s patterns of using SM^(35)^. Thus, future campaigns directed at female youth should place more emphasis on the SM platform(s) (such as Instagram) that attract the most attention from the target audience combined with YouTube for longer videos (e.g., cooking videos) and a website for more detailed content. Notably, a SM campaign does facilitate communication among adolescent peers and thus might able to shape social norms in the targeted population^(13)^.

With respect to campaign content, the information presented by the campaign was appropriate for the audience; it addressed adolescent girls’ health needs and was trustworthy, memorable and easy to understand. The decision to use key opinion leaders and a nutritionist as the source person for the campaign helped the campaign increase engagement, as studies have shown that the source of content was also cited as a variable impacting initial interest and interaction in SM content^(36)^.

However, more positive framing is encouraged, as respondents noted that adolescents are already prone to anxiety. Thus, a fear-based campaign should be avoided, although health warnings coupled with positive graphic messages may be the most effective way to promote healthier dietary habits^(37)^. Negative framing for obesity, for example, has not been shown to be effective in changing behavioural intentions when overweight people are the main target audience^(38)^. Therefore, positive emotional appeals in health messages could play a more effective role in promoting healthy behaviour, and in addition, this approach is also more ethical^(39–41)^. A study on reading and transmitting a health-related article also showed that positive emotional responses to the health-related information as well as the information’s emotional evocativeness were positively associated with engagement^(42)^.

Furthermore, the study found that beauty as a result of a healthy diet is an important factor in motivating adolescent girls to accept the campaign message about healthy eating, particularly with respect to needing fruits and vegetables. However, those who design health promotion campaigns should acknowledge the unintended social implications of conveying this kind of message, and they should strive to avoid negative health consequences. Many nutrition campaigns aimed at women, particularly younger ones, highlight weight loss as a way to improve health and beauty. In this way, the food, fashion and cosmetic industries have contributed to harmful dieting practices among women^(43)^.

The study participants noted that the most prominent individual-level barrier to their making healthy food themselves was their persistently strong perception that healthy food is not as tasty as food containing high levels of salt, sugar and fat. Such perceptions have been reported widely in other studies^(36,44)^. Personal barriers, such as a lack of perceived self-efficacy, as well as interpersonal barriers, such as peer pressure and a lack of parental support for healthy eating habits, were also identified. These barriers are common in the adolescent demographic group^(37,38,42,45)^. The identified environmental barriers to change were related to the availability of healthy food in the home or school canteen as well as a lack of control over food at home or when going out with friends. These barriers are not unique to respondents of the current study^(39,40,45)^.

Thus, to produce more meaningful behaviour change, messages should help reduce barriers to action, emphasise improving self-efficacy and encourage the target audience to prepare an action plan to translate their intentions into behaviour. This could be accomplished by providing more practical message content such as weekly menu plans and simplified recipes that can be prepared with limited cooking facilities. Last but not the least, the messages could also highlight the use of inexpensive ingredients in the campaign recipes.

Aside from the content of the message, the message format is also important – visually interactive campaigns seem preferred by the target audience. Videos featuring dieticians demonstrating simple recipes and snacks and showcasing the increased nutritional value – and greater volume – of healthy foods compared with the junk foods popular in the community have helped improve community awareness of nutrition problems^(46)^.

The food environment has also been identified as an important barrier; thus, future campaigns need to address wider audiences and networks in order to shape social norms related to healthy eating and to ensure the availability of and access to healthier food^(41,47)^. In addition to helping adolescents cultivate personal self-efficacy, societal collaborations to create structural and interpersonal supports are encouraged as part of a wider strategy to complement the SM campaign^(48)^.

Finally, active involvement with SM requires considerable resources to respond to inaccurate information and to ensure a proactive SM presence as well as to continuously monitor and actively disseminate information, comparable with a traditional mass media campaign^(12)^.

### Study limitations

At present, due to the short time frame for the evaluation, the evidence for the acceptance of the SM campaign and its success is still limited to qualitative evidence. While the TAM was also developed to measure behavioural outcomes^(15,16)^, this qualitative evaluation explored only intentions to engage in healthier behaviours. Moreover, the study did not utilise information from groups that did not access the campaign effectively or that refused to access the campaign, which does not allow for comparative analysis between groups. However, as a qualitative study, it has provided useful insight on the acceptance of the campaign as well as on strategies for improvement.

## Conclusion

The #prettyandpicky SM campaign was successfully launched, and by most indications, it was well accepted by Indonesian urban adolescent females, motivating them to act to protect their health. There were several lessons learned for future nutritional campaigns aimed at this demographic group. For one, findings indicated the need for a more focused selection of communication channels emphasising the platform with the greatest ease of use and usefulness, which would improve the campaign’s reach and level of engagement. Increasing the frequency of posting in the designated page(s) was cited by study subjects as a potentially important factor in sustaining high levels of engagement. Finally, the study highlighted the need for collaboration with parents and community institutions such as schools or colleges to both expand the campaign’s reach and provide a more supportive environment to help female adolescents address the remaining barriers to adopting healthier eating habits.
